# Transcriptome analysis of differentially expressed circRNAs miRNAs and mRNAs during the challenge of coccidiosis

**DOI:** 10.3389/fimmu.2022.910860

**Published:** 2022-11-15

**Authors:** Xiaolan Chen, Zhijun Wang, Yangfeng Chen, Ibrahim Akinci, Wei Luo, Yibin Xu, Endashaw Jebessa, Damer Blake, Nick Sparks, Olivier Hanotte, Qinghua Nie

**Affiliations:** ^1^ Lingnan Guangdong Laboratory of Modern Agriculture & State Key Laboratory for Conservation and Utilization of Subtropical Agro-Bioresources, College of Animal Science, South China Agricultural University, Guangzhou, Guangdong, China; ^2^ Guangdong Provincial Key Lab of Agro-Animal Genomics and Molecular Breeding, and Key Laboratory of Chicken Genetics, Breeding and Reproduction, Ministry of Agriculture, Guangzhou, Guangdong, China; ^3^ School of Life Sciences, Chongqing University, Chongqing, China; ^4^ School of Life Sciences, University of Nottingham, Nottingham, United Kingdom; ^5^ Department of Animal Breeding and Genetics, Poultry Research Institute, Ankara, Turkey; ^6^ State Key Laboratory of Livestock and Poultry Breeding & Guangdong Key Laboratory of Animal Breeding and Nutrition, Institute of Animal Science, Guangdong Academy of Agricultural Sciences, Guangzhou, China; ^7^ LiveGene – CTLGH, International Livestock Research Institute (ILRI), Addis Ababa, Ethiopia; ^8^ Pathobiology and Population Sciences, Royal Veterinary College, North Mymms, United Kingdom; ^9^ Roslin Institute Building, Scotland’s Rural College, Edinburgh, United Kingdom

**Keywords:** avian coccidiosis, sasso chicken, circRNAs, *circMGAT5*, *MMD*

## Abstract

Avian coccidiosis is a common enzootic disease caused by infection of *Eimeria* species parasites. It causes huge economic losses in the global poultry industry. Current control using anticoccidial drugs or vaccination is limited due to drug resistance and the relatively high cost of vaccines. Improving host genetic resistance to *Eimeria* species is considered an effective strategy for improved control of coccidiosis. Circular RNAs (circRNAs) have been found to function as biomarkers or diagnoses of various kinds of diseases. The molecular biological functions of circRNAs, miRNAs, and mRNAs related to Sasso chicken have not yet been described during *Eimeria* species challenge. In this study, RNA-seq was used to profile the expression pattern of circRNAs, miRNAs, and mRNAs in spleens from *Eimeria tenella*-infected and non-infected commercial dual-purpose Sasso T445 breed chickens. Results showed a total of 40 differentially expressed circRNAs (*DEcircRNAs*), 31 differentially expressed miRNAs (*DEmiRNAs*), and 820 differentially expressed genes (*DEmRNAs*) between infected and non-infected chickens. Regulatory networks were constructed between differentially expressed circRNAs, miRNAs, and mRNAs to offer insights into the interaction mechanisms between chickens and *Eimeria* spp. Functional validation of a significantly differentially expressed circRNA, *circMGAT5*, revealed that *circMGAT5* could sponge *miR-132c-5p* to promote the expression of the *miR-132c-5p* target gene *monocyte to macrophage differentiation-associated* (*MMD*) during the infection of *E. tenella* sporozoites or LPS stimulation. Pathologically, knockdown of *circMGAT5* significantly upregulated the expression of macrophage surface markers and the macrophage activation marker, *F4/80* and *MHC-II*, which indicated that *circMGAT5* might inhibit the activation of macrophage. *miR-132c-5p* markedly facilitated the expression of *F4/80* and *MHC-II* while *circMGAT5* could attenuate the increase of *F4/80* and *MHC-II* induced by *miR-132c-5p*, indicating that *circMGAT5* exhibited function through the *circMGAT5*-*miR-132c-5p*-*MMD* axis. Together, our results indicate that circRNAs exhibit their resistance or susceptive roles during *E. tenella* infection. Among these, *circMGAT5* may inhibit the activation of macrophages through the *circMGAT5*-*miR-132c-5p*-*MMD* axis to participate in the immune response induced by *Eimeria* infection.

## Introduction

Avian coccidiosis is a common enzootic disease caused by infection of seven *Eimeria* species (*E. acervulina, E. brunetti, E. maxima, E. mitis, E. necatrix, E. praecox, and E. tenella*). All seven species of *Eimeria* infect the gastrointestinal tract in a site-specific manner. Among which, *E. tenella* specifically infect the caeca part with high pathogenicity and high mortality, resulting in inefficient feed utilization, impaired growth rate, and reduced egg production (1). Therefore, *Eimeria* infection seriously impairs the chicken’s health and productivity, causing huge losses to the poultry industry (2). Current prevention and control methods for coccidiosis primarily depend on the careful use of anticoccidial drugs or vaccination (3–5). However, widespread drug resistance, high parasite prevalence and environmental persistence can still cause the outbreaks of coccidiosis (3). Drug resistance, public and legislative concerns regarding drug use and residues in livestock production, and the relatively high cost of vaccines are driving demand for novel alternatives for long-term control of chicken coccidiosis (6, 7). Selective breeding of chickens to improve resistance to coccidiosis is one possible strategy, screening to identify genes that contribute to resistance or susceptibility to coccidiosis.

Immune response to coccidiosis is associated with gut-associated lymphoid tissues (GALT), mucosal-associated lymphoid tissues (MALT), spleen, thymus, peripheral blood, bursa of Fabricius, and intestine. They either provides physical barrier or a complex set of cell-mediated immune (CMI) response. CMI response are regulated by T lymphocytes, macrophages, and natural killer (NK) cells through various tissues (8). For example, in the spleen, Th1 immune response and NK cell response was demonstrated in the *Eimeria*-infected chickens. Th1 immune response was enhanced and NK cells presence was involved in regulating IFN-γ secretion. Additionally, various cytokines and chemokines have been characterized to being differently expressed in spleen, including IL-6, IL-8, and C-C motif chemokine ligand 2 (CCL2), IL-1β, IL-10, IFN-γ, and tumor necrosis factor (TNF)-α (9, 10). They exhibit their specific role in host immunoregulation during primary or secondary infection. For example, IFN-γ is considerably expressed in the spleen and cecal tonsil and directly inhibits the development of *Eimeria* (11) and IL-10 plays a significant role in downregulating harmful inflammatory responses (10). However, the directly immune response genes involved in regulating chicken coccidiosis remain largely unknown.

Circular RNAs (circRNAs) represent a class of covalently-closed RNA molecules with diverse functional mechanisms, including sponging miRNAs, interacting with RNA binding proteins, forming R-loop and translating functional proteins (12–16). Among which, acting as miRNA sponge is a well-studied function of circular RNA, also known as a competing endogenous RNA mechanism (ceRNA) (17). The ceRNA mechanism is that messenger RNAs, transcribed pseudogenes, and long noncoding RNAs competitively combine with the same miRNA response elements, and then eliminate the inhibition of miRNA on their target genes (18). Various circRNAs have been found to be involved in modulation of immune responses to disease processes (19, 20), viral infection (21), as well as various avian epidemic diseases (22), resulting in different pathological phenotypes. However, the functional roles of circRNAs in response to avian coccidiosis remain largely unknown.

*Eimeria tenella* are one of the most economically important parasites that infect chickens (23). In order to explore circRNAs responses to *E. tenella* infection in commercial dual-purpose Sasso T445 chickens, we performed whole transcriptome sequencing of spleen tissues to study expression pattern and functional validation of potential circRNAs.

## Materials and methods

### Ethics statement

The challenge experiment was carried out at the ILRI (International Livestock Research Institute) poultry research facility in Addis Ababa, Ethiopia. The protocol was approved by the ILRI (International Livestock Research Institute) IACUC committee number with the reference IACUC-RC2019-01.

### Coccidiosis challenge experiment

*Eimeria tenella* oocysts of the reference Houghton strain (24) were amplified and purified as described previously (25) to challenge Sasso T445 chickens. Commercial one day old Sasso T445 chicks were obtained from a commercial company (EthioChicken) and divided into control (NSF, non-infected Sasso-first collection) and infected (ISF, infected Sasso-first collection) groups with 24 chickens in each group. After rearing for 21 days with water and *ad lib* feed in separate coccidia-free cages, each Sasso T445 chicken in the ISF group was orally inoculated with 10000 sporulated oocysts. The same volume of distilled water was inoculated to each Sasso chicken in the NSF group. The experimental site was fully environmental controlled (Closed) Houses. Temperature and humidity were established and followed according to the management guide by Cobb 500 (26).

### Tissue sample collection and lesion scoring

Spleen tissues were collected immediately post-mortem from 6 chickens in each group 4 days post-infection and stored in RNA later (Qiagen, Hilden, Germany).

Lesion scoring was performed according to the scoring technique of Johnson and Reid (27).

### RNA isolation, complementary DNA synthesis, and quantitative real-time PCR

Total RNA was isolated using TRIzol reagent (TaKaRa, Otsu, Japan). For circRNA and mRNA, cDNA synthesis was performed using the PrimeScript RT reagent kit with genomic DNA (gDNA) eraser (perfect real time) (TaKaRa, Otsu, Japan). The reverse transcription reaction for miRNA was performed using ReverTra Ace qPCR RT Kit (Toyobo, Osaka, Japan). Bulge-loop primers were synthesized by Ribobio (Guangzhou, China) for miRNAs. Quantitative real-time PCR with an iTaq Universal SYBR Green supermix kit (Bio-Rad, USA) and analyses with the 2^- ΔΔCt^ method were performed as described previously (28). The *β-actin* gene was used as a reference gene for circRNAs and mRNAs, and U6 snRNA was used as a reference gene for miRNAs. The sequences of all primers were provided in **Supplementary Table 1**.

### RNA sequencing

After spleen tissue RNA was isolated using TRIzol reagent (TaKaRa, Otsu, Japan), the quantity and quality of RNA were evaluated by agarose gel electrophoresis and Nanodrop. The RNA integrity number (RIN) was determined by Agilent 2100 Bioanalyzer, and a total amount of 3 µg RNA per sample with a RIN value of ≥ 7 were subjected to subsequent sequencing analysis. Before RNA library construction, ribosomal RNA (rRNA) was removed from the total RNA using Epicentre Ribo-Zero™ rRNA Removal Kit (Epicentre, Madison, Wisconsin, USA) following the manufacturer’s instructions. Surplus RNA was subjected to library construction without RNase R digestion. Sequencing libraries were generated using NEBNext^®^ Ultra™ RNA Library Prep Kit for Illumina^®^ and index codes were added to attribute sequences to each sample. Subsequently, high-throughput RNA-seq was performed on the Illumina HiSeq 2000 platform (Illumina, San Diego, CA, USA). The raw Illumina sequencing reads were cleaned by removing empty reads, adapter sequences, reads with over 10% N sequence, and low-quality reads through in-house perl scripts. At the same time, Q20, Q30 and GC content of the clean data were calculated. All downstream analyses were based on the cleaned high-quality data. Filtered reads were mapped to the chicken reference genome *Gallus gallus* 6 (ftp://ftp.ensembl.org/pub/release-96/fasta/gallus_gallus/dna/) using HISAT (29). Mapped reads were assembled and transcripts were constructed using StringTie (30).

miRNA sequencing (miRNA-seq) libraries were constructed by the following steps: 1) 3′-adaptor ligation; 2) 5′-adaptor ligation; 3) cDNA synthesis performed using Illumina real-time primers and amplification primers; 4) PCR amplification; and 5) size selection of 135–155 bp PCR-amplified fragments (corresponding to ~15–35 nt small RNAs). After library construction, the quality and concentration of the sequencing library were assessed by Agilent 2100 prior to sequencing on an Illumina HiSeq 2000 platform (Illumina, San Diego, CA, USA) according to the manufacturer’s instructions. Raw reads were cleaned by removing empty reads, adapter sequences, reads with over 10% N sequence, low-quality reads and polyA/T/G/C reads through in-house perl scripts. At the same time, Q20, Q30 and GC content of the clean data were calculated. All the downstream analyses were based on the cleaned high-quality data. Filtered reads were mapped to the chicken reference genome *Gallus gallus* 6 (ftp://ftp.ensembl.org/pub/release-96/fasta/gallus_gallus/dna/) using Bowtie (31). The mapped reads were then compared using blast with the specific sequence in miRbase to identify annotated miRNA. miREvo (32) and mirdeep2 (33) were used for novel miRNA predication.

### Profiling circRNAs, miRNAs, and mRNAs in Sasso chickens

Find_circ (34) and CIRI2 (35–37) were utilized for identification of circRNAs in this study. CircRNAs identified by both methods and with at least 2 reads were considered for further analysis. CircRNAs identified in each sample were quantified as TPM (copy number of transcripts per million) and differentiation analysis was performed by Ballgown (38). The cutoff for differentially expressed circRNAs was *P* < 0.05 and | (fold change) | > 0.

For miRNA, TPM was used to measure the expression of miRNAs and DESeq2 was used for *DEmiRNA* analysis with a cut off of *P* < 0.05 and | (fold change) | > 0.

The expression abundance of mRNAs was calculated using fragments per kb per million reads (FPKM). The cutoff for differentially expressed mRNAs was *P* < 0.05 and | (fold change) | > 0.

### Construction of circRNA-miRNA, miRNA-mRNA, and circRNA-miRNA-mRNA networks

All circRNAs were used to predict miRNAs potential binding sites using miRanda and all negatively expressed *DEcircRNA*-*DEmiRNA* pairs with threshold parameters single-residue-pair match scores> 140, ΔG< -10 kcal/mol. For the construction of miRNA-mRNA networks, we selected all potential negatively expressed *DEmiRNA-DEmRNA* pairs predicted by miRDB (http://mirdb.org/). Based on the negative *DEcircRNA*-*DEmiRNA* and *DEmiRNA*-*DEmRNA* pairs, we construct the ceRNA networks of *DEcircRNA*-*DEmiRNA*-*DEmRNA* to better understand the regulatory networks of circRNA, miRNA, and mRNA in response to *E. tenella* infection. All networks were generated using Cytoscape 3.7.2 (39).

### RNA oligonucleotides and plasmid construction

siRNAs targeted to *circMGAT5* (si-circFGFR2, 5′-AGCTTAATGTAGCAGGATG-3′) or a non-specific siRNA negative control, *miR-132c-5p* mimic, mimic control duplexes, *miR-132c-5p* inhibitor, inhibitor control, the 3′ end biotinylated *miR-132c-5p* mimic (AGCCAUGACUGUAGACUGUUACU) and control duplexes used in this study were synthetized by RiboBio (Guangzhou, China).

For *circMGAT5* overexpression plasmid construction, the linear sequence of *circMGAT5* was amplified and cloned into the pCD25-ciR expression vector. For pmirGLO-*circMGAT5*-wild/pmirGLO-*circMGAT5*-mutant reporter and pmirGLO-*MMD*-wild/pmirGLO-*MMD*-mutant reporter construction, the corresponding wild sequence and the mutant sequence were synthetized by Tsingke Biotechnology (Beijing, China) and then cloned into the pmirGLO expression vector.

### Cell culture and transfection

Chicken embryo fibroblast cell line (DF-1) cells were cultured in high-glucose Dulbecco’s modified Eagle’s medium (Gibco, Grand Island, NY, USA) with 10% (v/v) fetal bovine serum (FBS) (Gibco, Grand Island, NY, USA) and 0.2% penicillin/streptomycin (Invitrogen, Carlsbad, CA, USA). Transfections were performed with Lipofectamine 3000 reagent (Invitrogen, Carlsbad, CA, USA) according to the manufacturer’s instruction. Nucleic acids were diluted in OPTI-MEM Medium (Gibco, Grand Island, NY, USA).

### Dual-luciferase reporter assay and miRNA pull down assay

To investigate the binding sites of *circMATG5* with *miR-132c-5p*, DF-1 cells were seeded in 96-well plates and then co-transfected with 100 ng of pmirGLO-*circMGAT5*-wild/pmirGLO-*circMGAT5*-mutant reporter, and 50 nM of *miR-132c-5p* mimic or mimic control duplexes by using Lipofectamine 3000 reagent (Invitrogen, Carlsbad, CA, USA). Similarly, to explore the target relationship of *miR-132c-5p* and *MMD*, 100 ng of pmirGLO-*MMD*-wild/pmirGLO-*MMD*-mutant reporter, and 50 nM of *miR-132c-5p* mimic or mimic control duplexes were co-transfected in DF-1 cells in 96-well plates. After 48 h post-transfection, luciferase activity analysis was performed using a Fluorescence/Multi-Detection Microplate Reader (BioTek, Winooski, VT, USA) and a Dual-GLO^®^ Luciferase Assay System Kit (Promega, Madison, WI, USA). Firefly luciferase activities were normalized to Renilla luminescence in each well.

For miRNA pull down assay, 100 nM of 3′ end biotinylated *miR-132c-5p* mimic and control duplexes (RiboBio, Guangzhou, China) were transfected into HD11 cells. At 48 h after transfection, the cells were harvested and washed in PBS, then lysed in lysis buffer. A total of 40 µl washed streptavidin magnetic beads were blocked for 2 h and used to pull down the biotin-coupled RNA complex. Before RNA complex pulldown, 100 µl lysed cells were taken to extract the input RNA. For pulldown reactions, lysed cells and blocked streptavidin magnetic beads were incubated for 4 h on a rotator at a low speed (20 rpm/min). The beads were washed with washing buffer five times. After the wash steps, elution buffer and phenol-chloroform-isopentyl alcohol mixture (25:24:1) were used to harvest the biotin-coupled RNA complex. Finally, the abundance of *circMGAT5* in each group was evaluated by qRT-PCR analysis.

### Purification and the infection of the *Eimeria tenella* sporozoite

For cell challenge experiments, sporulated *E. tenella* oocysts were presented by Associate Professor Ruiqing Lin from South China Agricultural University. For sporozoite purification sporulated *Eimeria tenella* oocysts were first cleaned with sterile PBS. Oocysts walls were disrupted by vortexing with glass beads, confirming disruption using a light microscope. Vortex steps were repeated to maximize the number of released sporocysts. The supernatant with released sporocysts was transferred to a new 15-ml tube and the glass beads were washed 2–3 times with sterile PBS to collect any remaining sporocysts. All collected sporocysts were washed with PBS three times, and then the washed sporocysts were enzymatically excysted with 0.25% trypsin (Biochrom, Germany) and 4% sodium taurocholic acid (Sigma, Germany) in sterile PBS at 41°C for 60 to 90 min. During the incubation, monitoring of excystation was performed every 30 min with light microscopy. After incubation, free sporozoites were washed with sterile PBS three times and filtered with a G3 funnel through a vacuum filtration step. After filtration, sporozoites were washed with sterile PBS three times to finish the purification protocol.

Prior to *E. tenella* sporozoite infection of HD11 cells, purified sporozoites were counted using a haemocytometer and adjusted to 1×10^5^ per 1 ml with cell culture medium containing 5% fetal bovine serum. Infection was performed by culturing HD11 cells with cell culture medium containing 5% serum and 1×10^5^ sporozoites per 1 ml.

### Statistical analysis

The values reported in each graph are expressed as the mean ± standard error of the mean (S.E.M.) of at least three independent experiments. Statistical details are provided in each figure legend. We considered *P* < 0.05 to be statistically significant. * *P* < 0.05; ** *P* < 0.01.

## Results

### Occurrence of intestinal lesions following the challenge of *E. tenella* in sasso chickens

48 one-day-old Sasso T445 chickens were divided into control (NSF, distilled water) and infected (ISF, 10000 sporulated oocysts per bird) groups with 24 chickens in each group. Caecal lesion scoring for each chicken in both ISF and NSF groups at day 4 post infection revealed an average lesion score of 2.5 ± 0.29 in the NSF group and 0.25 ± 0.25 in the ISF group (*P* < 0.01; **Supplementary Figure 1**).

### Identification of differentially expressed circRNAs, miRNAs, and mRNAs during *E. tenella* infection

**Figure 1:**
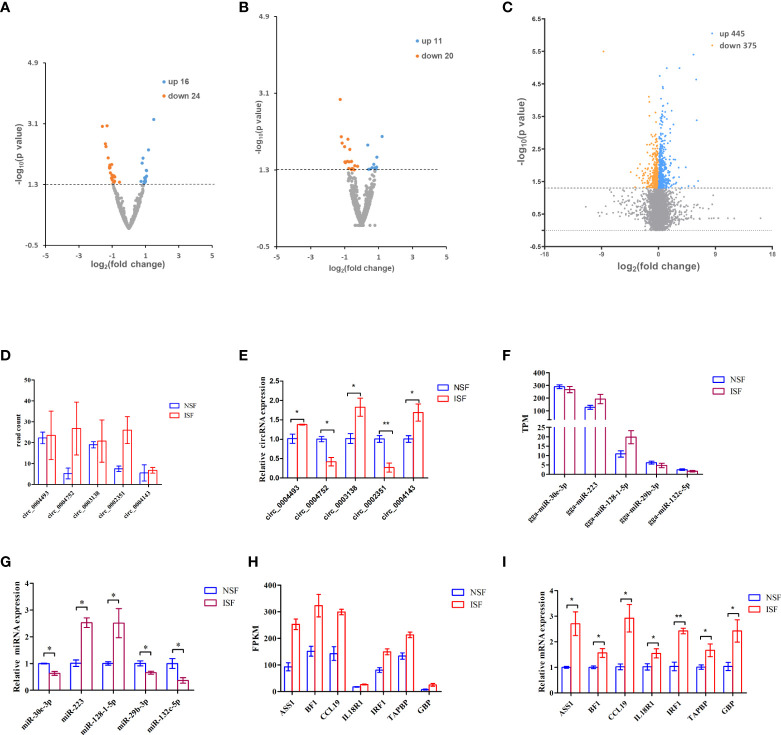
Volcano map of differentially expressed circRNAs, miRNAs and mRNAs associated with *E. tenella* infection. **(A)** Volcano map of differentially expressed *circRNAs*. **(B)** Volcano map of differentially expressed miRNAs. **(C)** Volcano map of differentially expressed mRNAs. For **(A-C)**, threshold used to define differentially expressed genes is(fold change)> 0 and *P* < 0.05. **(D)** Read count of the selected *DEcircRNAs* from the sequence data. **(E)** RT-qPCR quantification of the selected *DEcircRNAs*. **(F)** TPM of the selected *DEmiRNAs* from the sequence data. **(G)** RT-qPCR quantification of the selected *DEmiRNAs*. **(H)** FPKM of *DEmRNAs* from the sequence data. **(I)** RT-qPCR quantification of the selected *DEmRNAs*. For D-I, results are shown as mean ± SEM. Statistical significance of differences between means was assessed using unpaired Student’s t-test. (*P < 0.05; **P < 0.01).

To investigate circRNAs, miRNAs and genes involved in the regulation of avian coccidiosis, we performed RNA-seq and did differential expression analysis to screen candidate circRNAs, miRNAs and genes. The cutoff for differential expression of circRNAs, miRNAs or mRNAs was *P* < 0.05 and | (fold change) | > 0 (**Supplementary Excel 1–3**). A total of 40 differentially expressed *circRNAs* (*DEcircRNAs*, 16 upregulated and 24 downregulated) (**Figure 1A**), 31 differentially expressed miRNAs (*DEmiRNAs*, 11 upregulated and 20 downregulated) (**Figure 1B**), and 820 differentially expressed genes (*DEmRNAs*, 445 upregulated and 375 downregulated) (**Figure 1C**) were identified. To confirm the sequencing result, five *circRNAs* (including *circ_0004143*, named *circMGAT5* in this study), five miRNAs and seven genes were randomly selected to evaluate their expression level by qRT-PCR analysis. Results showed that the expression pattern of those selected *circRNAs* (**Figures 1D, E**), miRNAs (**Figures 1F, G**) and genes (**Figures 1H, I**) in the NSF and ISF groups were in line with the sequencing results. Additionally, the products of the selected DEcircRNAs were validated by Sanger sequence and agarose gel in **Supplementary Figure 2** (the result of *circ_0004143* was shown in **Figure 5A**), and the results demonstrated their circular structures.

### Interaction networks of circRNA-miRNA, miRNA-mRNA, and circRNA-miRNA-mRNA

**Figure 2:**
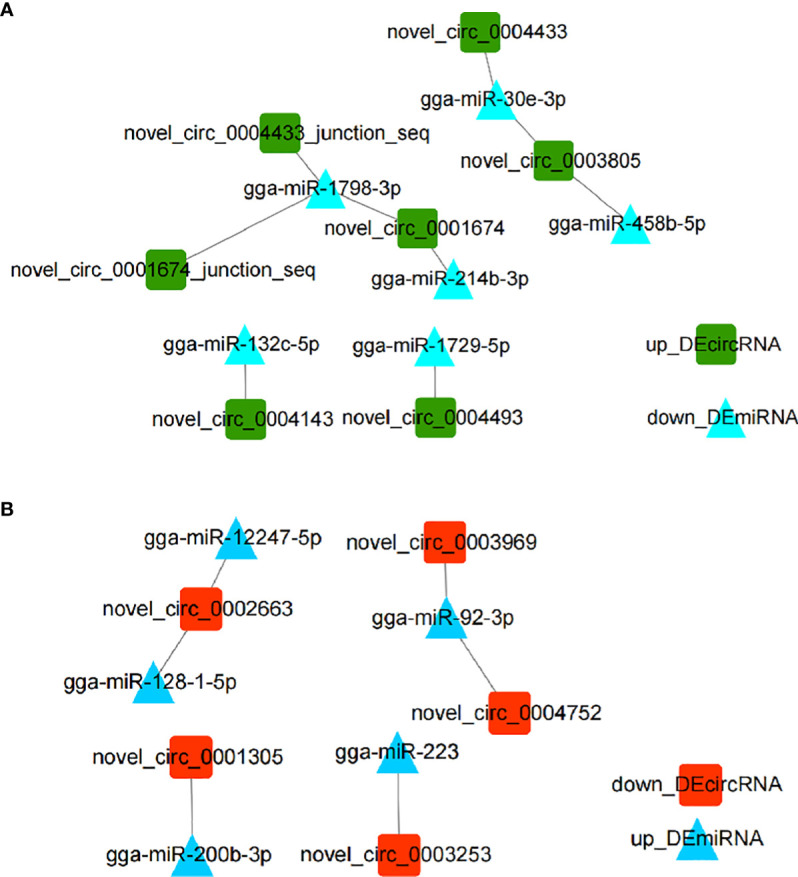
Interaction networks of *DEcircRNA*-*DEmiRNA* pairs involved in the immune response to *E. tenella* infection with threshold parameters (single-residue-pair match scores> 140, ΔG< -10 kcal/mol). **(A)** Networks of *up*_*DEcircRNA*-*down*_*DEmiRNA* generated using Cytoscape 3.7.2. **(B)** Networks of *down*_*DEcircRNA*-*up*_*DEmiRNA* generated using Cytoscape 3.7.2.

CircRNAs have been widely investigated as miRNA sponges participating in regulation of responses to various infectious diseases (22). In order to investigate *DEmiRNAs* potentially regulated by *DEcircRNAs*, we performed *DEcircRNA*-*DEmiRNA* binding analysis through miRanda. Results showed that nine *up_DEcircRNA*-*down_DEmiRNA* pairs (**Figure 2A**) and six *down_DEcircRNA*-*up_DEmiRNA* pairs (**Figure 2B**) were obtained (**Figure 2** and **Supplementary Excel 4**). From the identified set *miR-1798-3p* was predicted to interact with three circRNAs, while other miRNAs were potentially associated with one or two circRNAs.

**Figure 3:**
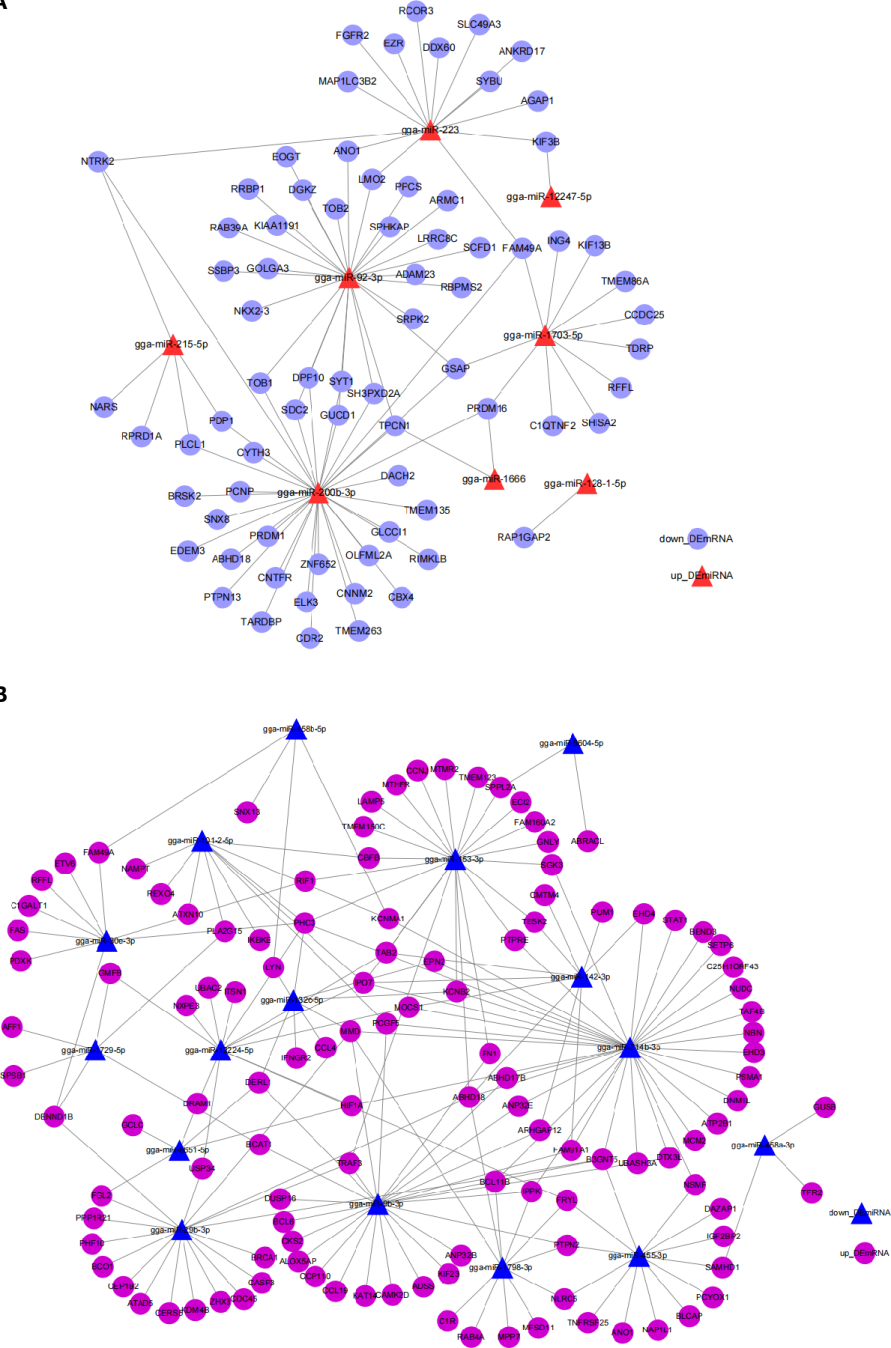
Networks of *DEmiRNA*-*DEmRNA* involved in the immune response to *E. tenella* infection predicted by miRDB. **(A)** Networks of *up*_*DEmiRNA*-*down*_*DEmRNA* generated using Cytoscape 3.7.2. **(B)** Networks of *down*_*DEmiRNA*-*up*_*DEmRNA* generated using Cytoscape 3.7.2.

miRNA can regulate gene expression by targeting the 3’UTR of mRNA, inhibiting their target either through degrading the mRNA or suppressing their translation. To better understand the potential function and functional mechanism of miRNA in response to *E. tenella* infection, we utilized the miRDB website to predict potential *DEmiRNA*-*DEmRNA* interaction pairs with opposed expression patterns during *E. tenella* infection. Ninety-seven *up_DEmiRNA*-*down_DEmRNA* interaction pairs (**Figure 3A**) and 192 *down_DEmiRNA*-*up_DEmRNA* (**Figure 3B**) negatively correlated pairs were predicted (**Figure 3**, and **Supplementary Excel 5**), suggesting a great diversity of miRNA in regulating *E. tenella* infection. *miR-200b-3p*, *miR-92-3p*, *miR-223*, *miR-214b-3p*, *miR-153-3p*, *miR-29b-3p* and *miR-455-3p* occupied the center of their networks, implying important roles in response to *E. tenella* infection.

**Figure 4:**
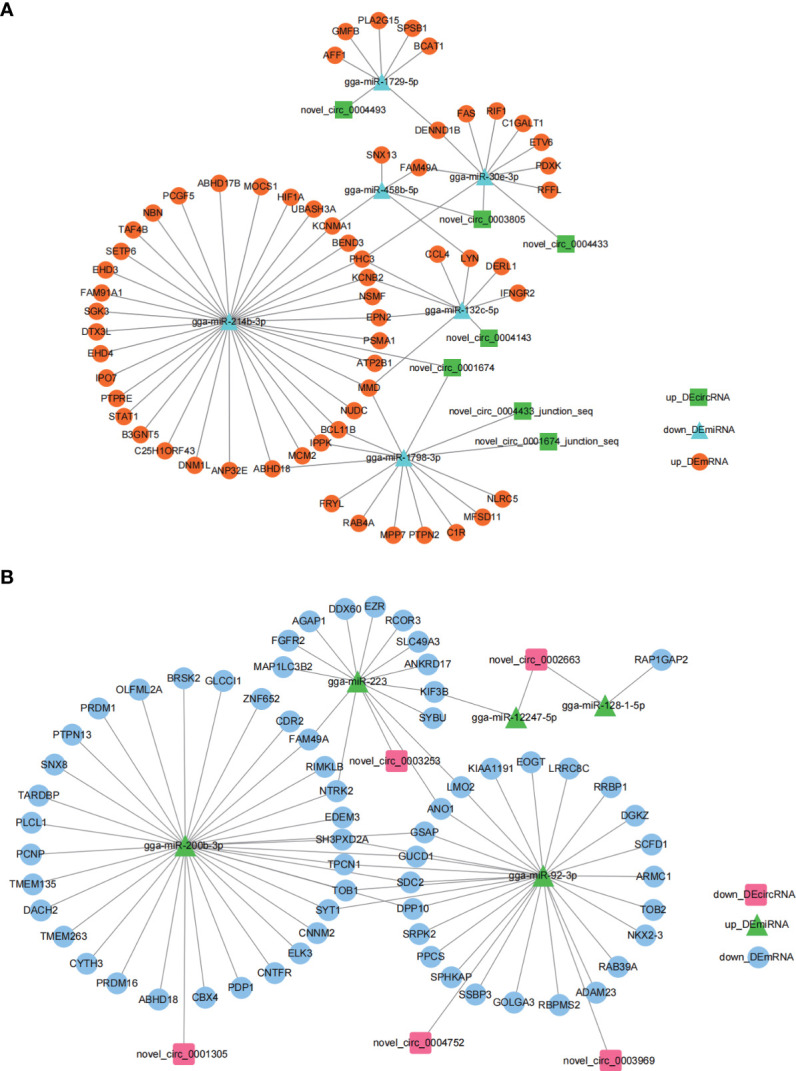
*DEcircRNA*-*DEmiRNA*-*DEmRNA* ceRNA regulatory networks generated by Cytoscape 3.7.2. **(A)** *up_DEcircRNA*-*down_DEmiRNA*-*up_DEmRNA* ceRNA networks generated using Cytoscape 3.7.2. **(B)** *down_DEcircRNA*-*up_DEmiRNA*-*down_DEmRNA* ceRNA networks generated using Cytoscape 3.7.2.

To explore the regulatory roles of circRNAs we constructed circRNA-miRNA-mRNA networks using Cytoscape 3.7.2. The miRNA sponge role of circRNA indicates a negative expression pattern and functional role of miRNA with its associated circRNA and mRNA. Based on this, we selected the *up_DEcircRNA*-*down_DEmiRNA*-*up_DEmRNA* axis (**Figure 4A**) and *down_DEcircRNA-up_DEmiRNA*-*down_DEmRNA* ceRNA axis (**Figure 4B**) to present ceRNA networks (**Figure 4** and **Supplementary Table 2**). *miR-214b-3p*, *miR-200b-3p* and *miR-92-3p* associated networks constituted the majority of the circRNA-miRNA-mRNA networks, suggesting an important role for these ceRNA networks in regulating *E. tenella* infection.

### Identification of *circMGAT5- miR-132c-5p-MMD* regulatory network

Previously, we confirmed that *MMD*, also a *DEmRNA* in this study, was associated with macrophage activation and differentiation (40). We inferred that certain *DEcircRNAs* were functional as miRNA sponges to promote *MMD* expression. Our results in **Figure 4**, support a putative interaction between *circMGAT5* (*circ-0004143*), *miR-132c-5p*, and *MMD*, indicating a potential epigenetic regulatory network (*circMGAT5*-*miR-132c-5p*-*MMD*) involved in the response to *E. tenella* infection. Therefore, we selected *circMGAT5 and miR-132c-5p* for further functional validation.

**Figure 5:**
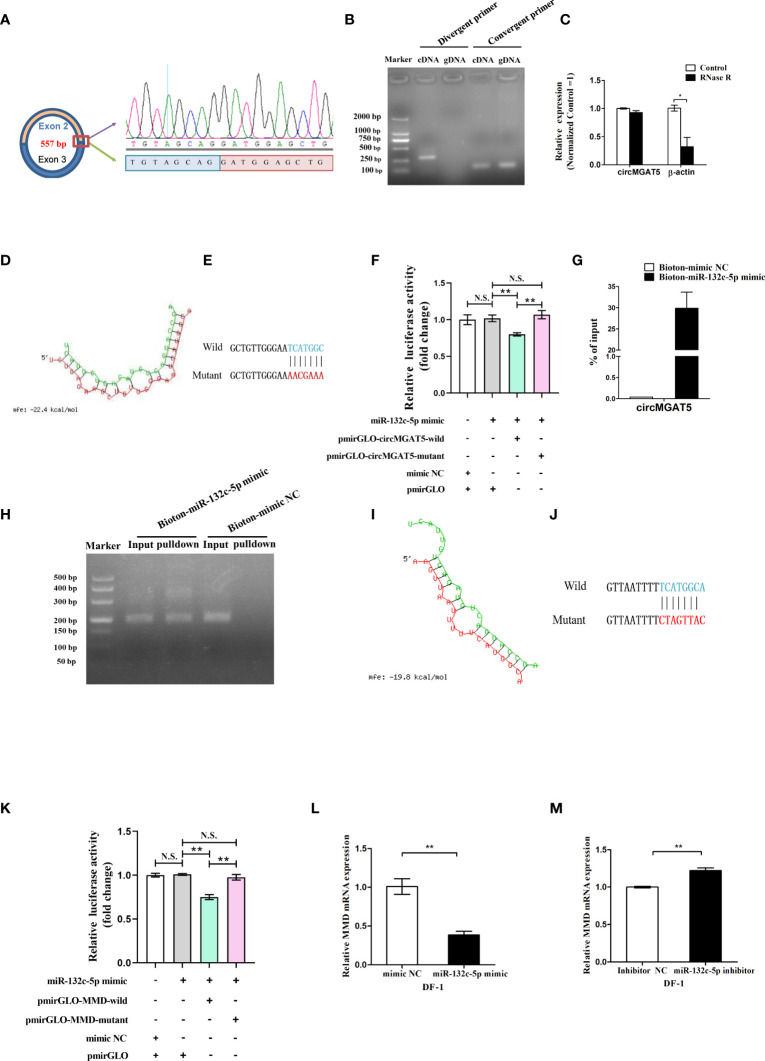
Identification of *circMGAT5*-*miR-132c-5p*-*MMD* axis. **(A)** Amplification of the junction sequence of *circMGAT5*. **(B)** Divergent primers amplify *circMGAT5* in cDNA but not genomic DNA (gDNA). **(C)** RT-qPCR quantification of *circMGAT5* after RNase R treatment. **(D)** The potential interaction model between *circMGAT5* and *miR-132c-5p* predicted by RNAhybird. **(E)** The wild and mutant binding site sequence between *circMGAT5* and *miR-132c-5p* in the pmirGLO vector. **(F)** Dual-luciferase reporter assay measuring the binding of *circMGAT5* to *miR-132c-5p*. **(G)** RT-qPCR quantification of *circMGAT5* enriched in the biotinylated *miR-132c-5p* mimic pull down RNA. **(H)** qPCR product of *circMGAT5* in the biotinylated *miR-132c-5p* mimic pull down RNA and biotinylated mimic NC. **(I-M)** *miR-132c-5p* interacts with *MMD*. **(I)** The potential interaction model between *miR-132c-5p* and *MMD* predicted by RNAhybird; **(J)** The wild and mutant binding site sequence between *miR-132c-5p* and *MMD* in the pmirGLO vector; **(K)** Dual-luciferase reporter assay measuring the binding of *miR-132c-5p* to *MMD*; **(L)** *miR-132c-5p* mimic inhibits the expression of *MMD*; **(M)** *miR-132c-5p* inhibitor promotes the expression of *MMD*. For **(C, F, G, K, L, M)**, results are shown as mean ± SEM. Statistical significance of differences between means was assessed using unpaired Student’s t-test. (*P < 0.05; **P < 0.01; N.S. no significant difference).

The *circMGAT5* sequence identified from the RNA-seq suggested it was generated from the second and the third *MGAT5* exons, represented by 557 bp (**Figure 5A** left). Based on this, the junction sequence of *circMGAT5* was identified with divergent and convergent primers (**Figure 5A** right and **B**). In addition, expression of *circMGAT5* was found to be stable after RNase R digestion, further confirming the circular structure of *circMGAT5* (**Figure 5C**).

Based on the hypothesized *circMGAT5*-*miR-132c-5p*-*MMD* axis, *circMGAT5* was predicted to interact with *miR-132c-5p*. To address the relationship between *circMGAT5* and *miR-132c-5p*, we utilized RNAhybrid to predict the binding site between *circMGAT5* and *miR-132c-5p* (**Figure 5D**). Subsequently, pmirGLO-*circMGAT5*-wild dual-luciferase and a pmirGLO-*circMGAT5*-mutant dual-luciferase reporters were generated by inserting the wild type (with the wild putative binding site) or mutant (with the mutational putative binding site) linear *circMGAT5* sequence (**Figure 5E**) into the 3′ end of the *firefly* luciferase in the pmirGLO luciferase vector. Subsequently, either pmirGLO-*circMGAT5*-wild/pmirGLO-*circMGAT5*-mutant reporter was co-transfected with *miR-132c-5p* mimic or mimic control duplexes in DF-1 cells to check the relative luciferase activity. The relative luciferase activity in DF-1 cells was significantly decreased when *miR-132c-5p* mimics were co-transfected with pmirGLO-*circMGAT5*-wild reporter compared with the *miR-132c-5p* mimic and their correspondent mutant reporter co-transfected group (**Figure 5F**). And the relative luciferase activity in DF-1 cells of *miR-132c-5p* mimic/mimic NC and pmirGLO co-transfected group, showed no difference with the *miR-132c-5p* mimic and pmirGLO-*circMGAT5*-mutant co-transfected group, suggesting the decreased luciferase activity in *miR-132c-5p* mimic and pmirGLO-*circMGAT5*-wild group was due to the binding between *miR-132c-5p* and *circMGAT5* but not and pmirGLO vector sequence. Taken together, a target relationship was confirmed between *circMGAT5* and *miR-132c-5p*.

To further confirm the interaction between *circMGAT5* and *miR-132c-5p*, we used a biotin-coupled miRNA pull down assay with a biotin-coupled *miR-132c-5p* mimic to address the endogenous binding relationship between them. Compared with the control fraction, we observed more than 25-fold enrichment of *circMGAT5* in the *miR-132c-5p* captured fraction (**Figures 5G, H**), demonstrating that endogenous *circMGAT5* could directly sponge *miR-132c-5p*.

In order to address the relationship between *miR-132c-5p* and *MMD*, we utilized RNAhybrid to predict the binding site between *miR-132c-5p* and *MMD* (**Figure 5I**). Subsequently, pmirGLO-*MMD*-wild and pmirGLO-*MMD*-mutant reporters were constructed by inserting the wild type (with the wild putative binding site) or mutant (with the mutational putative binding site) linear *MMD* sequence (**Figure 5J**) into the 3′ end of the *firefly* luciferase in the pmirGLO luciferase vector. Subsequently, the pmirGLO-*MMD*-wild or pmirGLO-*MMD*-mutant reporter was co-transfected with *miR-132c-5p* mimic or mimic control duplexes in DF-1 cells to check the relative luciferase activity. The relative luciferase activity in DF-1 cells was significantly decreased when *miR-132c-5p* mimics were co-transfected with pmirGLO-*MMD*-wild reporter compared with the *miR-132c-5p* mimic and their correspondent mutant reporter co-transfected group (**Figure 5K**). And the relative luciferase activity of *miR-132c-5p* mimic/mimic NC and pmirGLO co-transfected group showed no difference with the *miR-132c-5p* mimic and pmirGLO-MMD-mutant co-transfected group, suggesting the decreased luciferase activity in *miR-132c-5p* mimic and pmirGLO-MMD-wild group was due to the binding between *miR-132c-5p* and *MMD* but not and pmirGLO vector sequence. Moreover, overexpression of *miR-132c-5p* inhibited the expression of *MMD* (**Figure 5L**), while *miR-132c-5p* inhibition resulted in decreased *MMD* expression in DF-1 cells (**Figure 5M**). Thus, our results confirmed the target relationship between *miR-132c-5p* and *MMD*.

### *CircMGAT5* regulates macrophage activation and differentiation through the *circMGAT5-miR-132c-5p-MMD* axis

MMD was associated with macrophage activation and differentiation (40). To explore the role of *circMGAT5-miR-132c-5p-MMD* axis in macrophage activation and differentiation. We detected the effect of *miR-132c-5p* and *circMGAT5* on the expression of *MMD* during *E. tenella* sporozoite infection or LPS stimulation.

**Figure 6:**
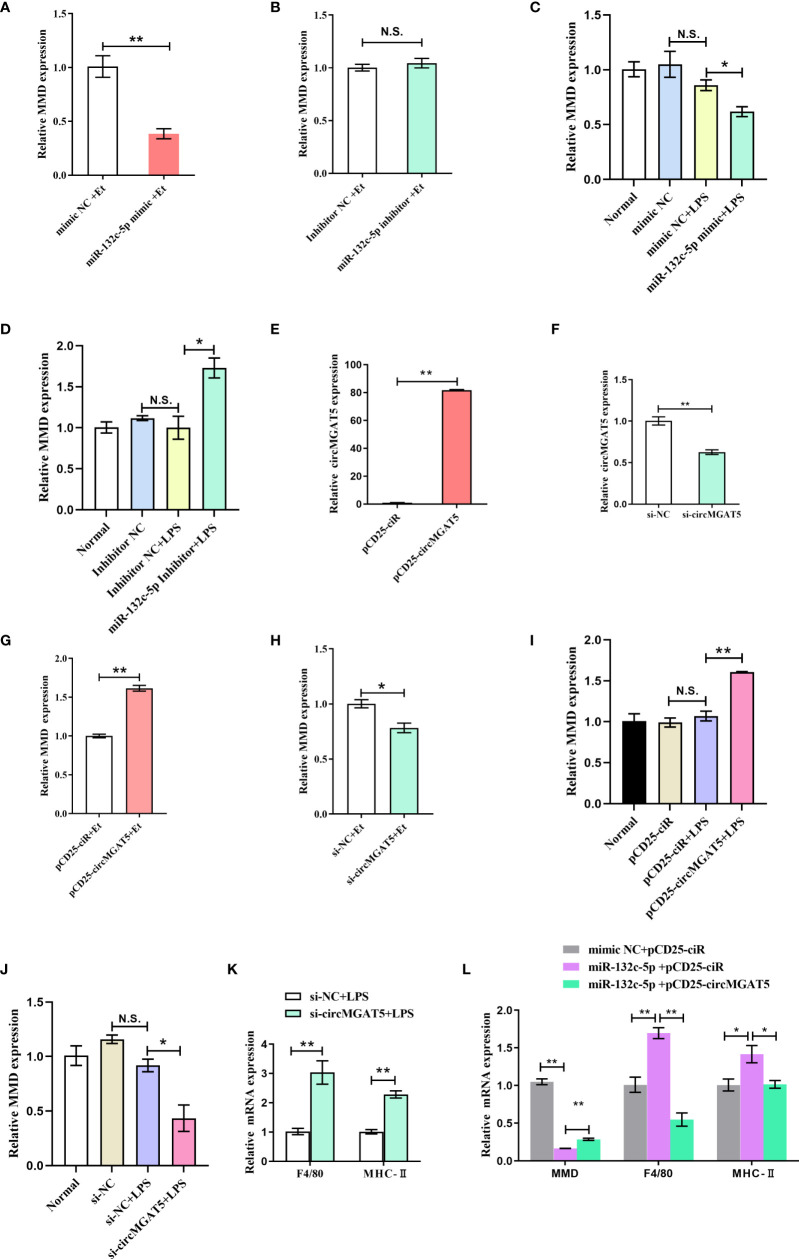
*circMGAT5* inhibits macrophage activation and differentiation through the *circMGAT5*-*miR-132c-5p*-MMD axis. **(A-D)** *miR-132c-5p* inhibits the expression of *MMD* during *E. tenella* infection or LPS stimulation in HD11 cells. **(A)** Expression of *MMD* was inhibited by *miR-132c-5p* mimic during *E. tenella* infection; **(B)** *miR-132c-5p* inhibitor promoted the expression of *MMD* during *E. tenella* infection; **(C)** Expression of *MMD* was decreased by *miR-132c-5p* mimic during LPS stimulation of HD11 cells; **(D)** *miR-132c-5p* inhibitor increased expression of *MMD* during LPS stimulation of HD11 cells. **(E-J)** *circMGAT5* promotes *MMD* expression during *E. tenella* sporozoite infection or LPS stimulation of HD11 cells. **(E)** Overexpression of *circMGAT5*. **(F)** Knockdown of *circMGAT5*. **(G)** Overexpression of *circMGAT5* promoted *MMD* expression during *E. tenella* sporozoite infection. **(H)** Knockdown of *circMGAT5* inhibited *MMD* expression during *E. tenella* sporozoite infection. **(I)** Overexpression of *circMGAT5* promoted *MMD* expression of during LPS stimulation of HD11 cells. **(J)** Knockdown of *circMGAT5* inhibited *MMD* expression during LPS stimulation of HD11 cells. **(K, L)** *circMGAT5* inhibits macrophage activation and differentiation through the *circMGAT5*-*miR-132c-5p*-MMD axis. **(K)** Knockdown of *circMGAT5* markedly increased *F4/80* and *MHC-II* expression, indicating that downregulation of *circMGAT5* may result in enhanced macrophage activation and differentiation. **(L)** *miR-132c-5p* markedly facilitated expression of *F4/80* and *MHC-II* while *circMGAT5* could attenuate the increase induced by *miR-132c-5p*. In addition, the expression of *MMD* in the *miR-132c-5p* and *circMGAT5* co-transfected groups was significantly higher than that in the *miR-132c-5p* overexpressing group, indicating that *circMGAT5* attenuates the inhibitory effect of *miR-132c-5p* on *MMD* and *circMGAT5* may inhibits macrophage activation and differentiation through the *circMGAT5*-*miR-132c-5p*-MMD axis. In all panels, results are shown as mean ± SEM. Statistical significance of differences between means was assessed using unpaired Student’s t-test. (*P < 0.05; **P < 0.01; N.S. no significant difference).

To explore the effect of *miR-132c-5p* on *MMD* expression, HD11 cells were transfected with mimic and inhibitor of *miR-132c-5p* for 6 h, and then subjected to *E. tenella* sporozoite infection or LPS stimulation. Expression of the *MMD* gene was detected 24 h after infection with *E. tenella* sporozoites. The results showed that *miR-132c-5p* mimic could significantly inhibit expression of the *MMD* gene during infection (**Figure 6A**), while *miR-132c-5p* inhibitor slightly promoted *MMD* expression, although not at a significant level (**Figure 6B**). Similarly, during the stimulation of LPS, *miR-132c-5p* significantly inhibited *MMD* expression (**Figure 6C**), while downregulation of *miR-132c-5p* significantly promoted the expression of *MMD* (**Figure 6D**) at the 6 h LPS post stimulation.

Similarly, to investigate the effect of *circMGAT5* on *MMD* expression, *E. tenella* sporozoite infection or LPS stimulation was performed after transfection of HD11 cells with *circMGAT5* overexpression vector or specific siRNA. The results showed that overexpression of *circMGAT5* (**Figure 6E**) significantly promoted *MMD* expression (**Figure 6G**) at 24 h post-infection, while interference with *circMGAT5* (**Figure 6F**) significantly inhibited the expression of *MMD* (**Figure 6H**). The same results were obtained at 6 h LPS post stimulation (**Figures 6I, J**), indicating that *circMGAT5* could promote expression of *MMD* during both *E. tenella* sporozoite infection or LPS stimulation.

Macrophages are important immune cells in the host. It was reported that *MMD* was preferentially expressed in mature macrophages and may affect the activation and differentiation of macrophages (41). Indeed, our previous work revealed that the knockdown of *MMD* upregulated expression of macrophage surface and activation markers, such as *F4/80* and *MHC-II* (42–44), indicating that *MMD* could inhibit macrophage activation and differentiation (40). Here, *circMGAT5* was able to elevate *MMD* expression suggesting that *circMGAT5* might play a potential role in macrophage activation and differentiation. To this end, we evaluated the expression of *F4/80* and *MHC-II* after knockdown of *circMGAT5* along with LPS stimulation. Results showed that knockdown of *circMGAT5* markedly increased *F4/80* and *MHC-II* expression, indicating that downregulation of *circMGAT5* may enhance macrophage activation and differentiation (**Figure 6K**). Moreover, in order to confirm *circMGAT5* function through the *circMGAT5*-*miR-132c-5p*-*MMD* axis, we co-transfected the *circMGAT5* overexpression plasmid/empty plasmid with *miR-132c-5p* and evaluated the expression of *MMD*, *F4/80* and *MHC-II* in each group. The result showed that *miR-132c-5p* facilitated expression of *F4/80* and *MHC-II*, and that *circMGAT5* could attenuate the increase induced by *miR-132c-5p* (**Figure 6L**). In addition, the expression of *MMD* in the *miR-132c-5p* and *circMGAT5* co-transfected group was significantly higher than that in the *miR-132c-5p* overexpressing group, indicating that *circMGAT5* attenuates the inhibitory effect of *miR-132c-5p* on *MMD* during LPS stimulation. Taken together, *circMGAT5* inhibits macrophage activation and differentiation through the *circMGAT5*-*miR-132c-5p*-MMD axis.

## Discussion

Avian coccidiosis causes serious intestinal disease in chickens, compromising productivity and animal welfare, resulting in huge economic losses every year (5, 45). Live attenuated and non-attenuated anticoccidial vaccines are effective tools to control and prevent (7), but the costs of production are high and capacity limited by the requirement for *in vivo* production. Selection of broiler chicken lines naturally resistant to coccidiosis may be an effective alternative strategy to control the effects and costs of coccidiosis (46, 47).

CircRNAs are molecules with a great diversity of functional mechanisms, including acting as miRNA sponges (17). In order to characterise the circRNAs involved in the immune response to coccidiosis, RNA-seq was performed to screen the candidate circRNAs and a total of 40 *DEcircRNAs* were identified. In line with previous studies, expression levels of circRNAs were altered by *Eimeria* challenge, although the number of differentially expressed circRNAs was small following different *Eimeria* species infection (*Eimeria necatrix* and *Eimeria tenella*) (48, 49). Functional annotation of *DEcircRNAs* in previous studies revealed that circRNAs are potentially involved in various immune related processes. These include adaptive immune responses with positive regulation of B cell activation and the B cell receptor signalling pathway, and the intestinal immune network for IgA production (48). In this study, the potential GO terms or KEGG pathways (**Supplementary Tables 3, 4**) enriched by the *DEcircRNAs* included telomere maintenance in response to DNA damage, membrane-bounded organelle, and nucleotide excision repair, which were not consistent with the previous report, indicating that the *DEcircRNAs* caused by different *Eimeria* species (*Eimeria necatrix* or *Eimeria tenella*) infection may carry different functions.

The 31 *DEmiRNAs* identified here were significantly enriched in transcription regulatory related terms (**Supplementary Table 5**). The immune-related KEGG pathways in the top 20 enriched KEGG pathways (**Supplementary Table 6**) included Intestinal immune network for IgA production, Notch signalling pathway, *Salmonella* infection, Influenza A, TGF-beta signalling pathway, and Herpes simplex infection, suggesting that the miRNAs identified play important roles in the immune response to coccidiosis. In this study, the *DEmRNAs* were significantly enriched in many immune related processes, such as immune response, immune system process, immune effector process, and cytokine-mediated signalling pathway (**Supplemental Tables 7, 8**). Among which, cytokine-mediated signalling pathway was also found to be enriched by the *DEmRNAs* caused by other *Eimeria* challenge process (50), which indicated that cytokine-mediated signalling pathway was one of the most important pathways in response for coccidiosis.

The regulatory networks of circRNA-miRNA, miRNA-mRNA, and circRNA-miRNA-mRNA showed that the *miR-214b-3p*, *miR-200b-3p*, and *miR-92-3p* associated networks occupied the majority of both *DEmiRNA*-*DEmRNA* and the *DEcircRNAs*-*DEmiRNAs*-*DEmRNAs* networks, suggesting an important role for these miRNAs-related ceRNA networks in regulating the *E. tenella* infection. Indeed, these miRNAs were involved in immunity and disease related processes. For example, miR-200b can participate in the infection process of chicken necrotizing enteritis disease (51). *miR-92-3p* is a potential biomarker for hepatocellular carcinoma (52) and non-small cell lung cancer screening (53); *miR-223* is extensively involved in various inflammatory responses or disease infection processes (54); *miR-153-3p* can be involved in regulating the occurrence and development of various tumors (55, 56). Our results show that they may also play an important role in the response to coccidiosis in poultry.

Previously, we confirmed that *MMD*, which was a *DEmRNAs* in this study, was associated with macrophage activation and differentiation (40). Acting as miRNA sponge is one of the most widely investigated functional mechanisms of circRNAs. A putative interaction between *miR-132c-5p* and *circMGAT5* (*circ_0004143*)/*MMD* was found by ceRNA networks analysis, indicating that *circMGAT5* may function through the *circMGAT5*-*miR-132c-5p*-*MMD* axis. A series of assays indicated the solid binding relationship between *miR-132c-5p* with *circMGAT5/MMD*. Pathologically, knockdown of *circMGAT5* significantly upregulated the expression of macrophage surface markers and the macrophage activation marker, *F4/80* and *MHC-II* after LPS stimulation, which indicated that *circMGAT5* may inhibit the activation of macrophage through inhibiting the expression of *MMD*, as knockdown of *circMGAT5* also resulted in reduced *MMD* expression. Meanwhile, *miR-132c-5p* markedly facilitated the expression of *F4/80*, *MHC-II*, and *circMGAT5* could attenuate the increase of *F4/80* and *MHC-II* induced by *miR-132c-5p*. indicating that *circMGAT5* is involved in activation of macrophages in response to *E. tenella* infection through the *circMGAT5*-*miR-132c-5p*-*MMD* axis.

Together, our study performed a comprehensive genomic analysis of the expression pattern, functional annotation and regulatory networks of the potential circRNAs, miRNAs, and mRNAs involved in the immune response to *E. tenella* infection, offering new insight into the mechanisms underlying interactions between *Eimeria* spp. and chicken. Moreover, we identified that the transcription of *circMGAT5* was significantly increased during *Eimeria* infection, and that it can inhibit the macrophage activation through the *circMGAT5*-*miR-132c-5p*-*MMD* axis, suggesting that *circMGAT5* can serve as a biomarker to diagnose chicken coccidiosis.

## Data availability statement

The data presented in the study are deposited in the China National Center for Bioinformation repository, accession number: CRA006601.

## Ethics statement

The animal study was reviewed and approved by the ILRI (International Livestock Research Institute) IACUC committee number with the reference IACUC-RC2019-01.

## Author contributions

XC and QN conceived and designed the study. XC carried out the majority of the experiments, analyzed the data, and draft the paper. ZW, YC, WL, EJ, and YX carried out part of the experiments. IA, OH and NS designed the challenge experiment and provided the chickens and the farm. DB provided the *E. tenella* oocysts for the on-station challenge the chickens. All authors contributed to the article and approved the submitted version.

## Funding

This work was supported by the Natural Scientific Foundation of China (31761143014), the Bill and Melinda Gates Foundation (BMGF) and with UK aid from the UK Government’s, Department for International Development (Grant Agreement OPP1127286) under auspices of the Centre for Tropical Livestock Genetics and Health (CTLGH), established jointly by the University of Edinburgh, SRUC (Scotland′s Rural College), and the International Livestock Research Institute The findings and conclusions contained within the paper are those of the authors and do not necessarily reflect positions or policies of the BMGF nor the UK Government.

## Acknowledgments

We would like to share our thanks to Ruiqing Lin from College of Veterinary Medicine, South China Agricultural University for his kindness donation of *Eimeria tenella* oocysts.

## Conflict of interest

The authors declare that the research was conducted in the absence of any commercial or financial relationships that could be construed as a potential conflict of interest.

## Publisher’s note

All claims expressed in this article are solely those of the authors and do not necessarily represent those of their affiliated organizations, or those of the publisher, the editors and the reviewers. Any product that may be evaluated in this article, or claim that may be made by its manufacturer, is not guaranteed or endorsed by the publisher.
