# Supplementary material for: Transcriptome analysis of differentially expressed circRNAs miRNAs and mRNAs during the challenge of coccidiosis
Source: Front Immunol. 2022 Nov 15;13:910860. doi: 10.3389/fimmu.2022.910860 (PMC9706185; doi:10.3389/fimmu.2022.910860)
Supplement: Supplementary file 6 [file DataSheet_1.doc]

**Supplementary Figures and Tables**

**Supplementary Figure 1**





**Coccidiosis causes weight loss and intestinal lesions in chickens.** The lesion scores of the caeca of the *Eimeria tenella* infected chickens were significantly higher than that of the control group at the 4 days of post infection.

**Supplementary Figure 2**

**
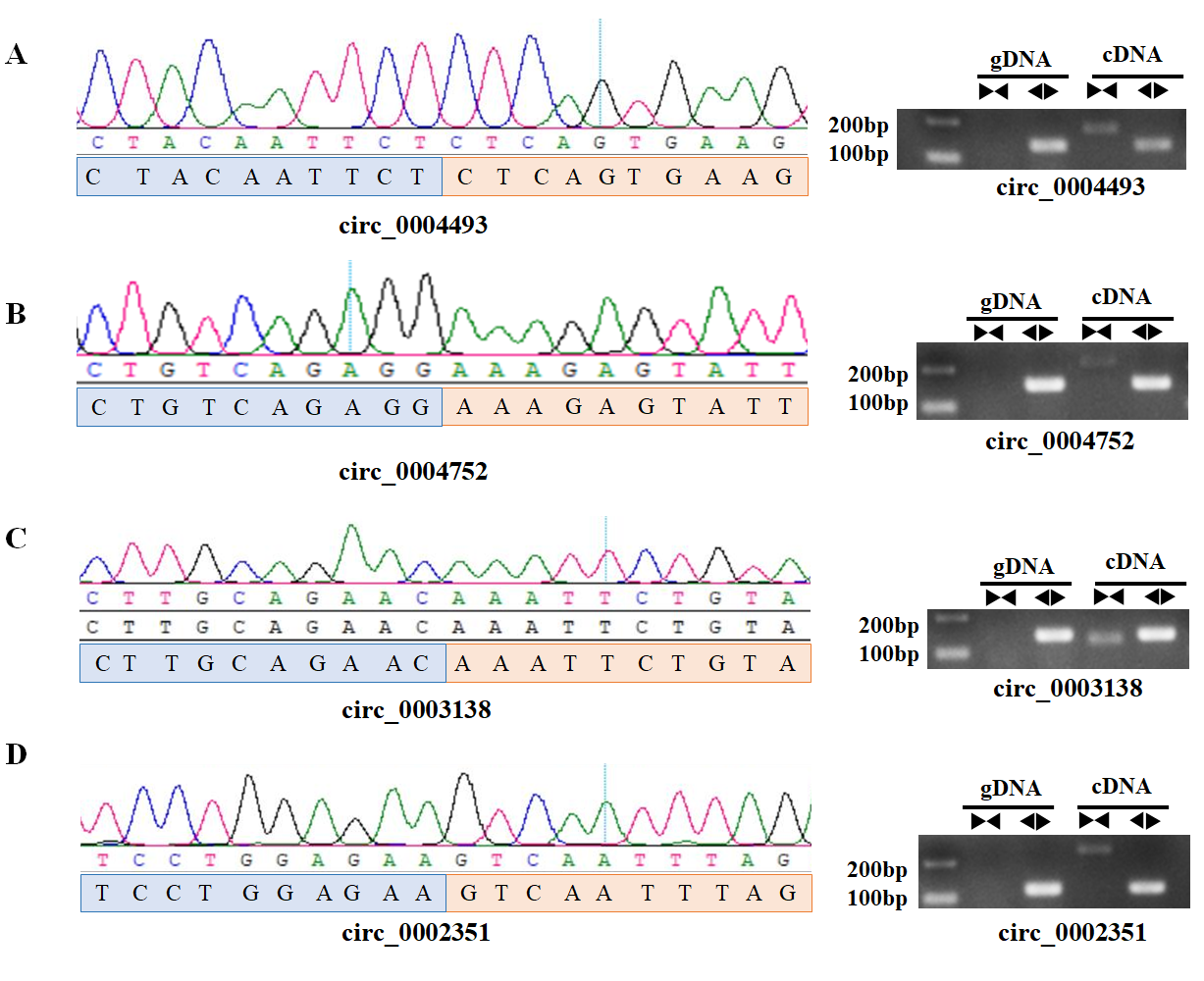
**

**Verification of the circular structure of the selected DEcircRNAs.** (A) The junction sequence of *circ_0004493* was verified by Sanger sequence and agarose gel. (B) The junction sequence of *circ_0004752* was verified by Sanger sequence and agarose gel. (C) The junction sequence of *circ_0003138* was verified by Sanger sequence and agarose gel. (D) The junction sequence of *circ_0002351* was verified by Sanger sequence and agarose gel.

**Supplementary table 1**

**Supplementary table 1 Primer sequence**

| Name | Primer sequence | | Length (bp) | Temperature (℃) | Use |
| --- | --- | --- | --- | --- | --- |
| circ_0004493 | F | CATGAAGAGAAAGCAGAACGAATAA | 214 | 56 | qPCR |
|  | R | AGAAAATCTGTCATAAGCCCAAGTC |  |  |  |
| circ_0004752 | F | TGGTCAAAGGCGGAGAGAAGCAT | 270 | 56 | qPCR |
|  | R | TCATTGGGGAAACCTCAGGGAGA |  |  |  |
| circ_0003138 | F | CAGCCTCTGTTTACTCTCA | 150 | 56 | qPCR |
|  | R | GCGTTTGTACTCCTGTTTT |  |  |  |
| circ_0002351 | F | CAGTATGTGCTTCACGAGG | 293 | 56 | qPCR |
|  | R | TTTCTGTGTTCTGGGGTCT |  |  |  |
| circ_0004143  (circMGAT5) | F | GGAACAGGAGCAAACTCTACCA | 293 | 56 | qPCR |
|  | R | ACCAAAAGTCACCAGAAAAAAG |  |  |  |
| 0004493-Con | F | TCCCAGTGAACCCTATCAAGG | 149 | 56 | PCR |
|  | R | CATGCTACGTTCAGCAATGG |  |  |  |
| 0004752-Con | F | GGTCCACATCAAACGACCCA | 180 | 56 | PCR |
|  | R | CTGTGAGGGCTGGCATTAGA |  |  |  |
| 0003138-Con | F | AGGACTCTGGATTTCGACCC | 163 | 56 | PCR |
|  | R | GGGAGGACACCTCTCCGAA |  |  |  |
| 0002351-Con | F | GACAGCAGGCTTCACCTCTT | 132 | 56 | PCR |
|  | R | AGGAAAGCGCCTGTTCAAAG |  |  |  |
| 0004143-Con | F | TTTTTCTGGTGACTTTTGGT | 134 | 56 | PCR |
|  | R | TGCTTTGATGTATCTTTTGC |  |  |  |
| ASS1 | F | GGTCACCAACACCAGGAATG | 120 | 58 | qPCR |
|  | R | ACAAAGCGGTTCTCCACAAT |  |  |  |
| BF1 | F | CACCAAGAGGAAATGGGAGG | 183 | 58 | qPCR |
|  | R | CGGCAGGACAAGGTCAGGAT |  |  |  |
| CCL19 | F | TGCCTGCGGACGAGCGAGAA | 209 | 58 | qPCR |
|  | R | TTGCCTTGATTTGGGACCTT |  |  |  |
| IL18R1 | F | AATGCAGTGATCTGTCTGTCA | 192 | 58 | qPCR |
|  | R | CCAGCCTGATTGTATTGGTGC |  |  |  |
| IRF1 | F | ATAAAGCTGCTCGAACCCAC | 159 | 58 | qPCR |
|  | R | CATGCGGAACTCCAACTCTG |  |  |  |
| TAPBP | F | GGGCACCTACATCTGCTCCG | 115 | 58 | qPCR |
|  | R | AGCCACCACCAGGTTCTTCG |  |  |  |
| GBP | F | CAACAACCCAGCATCCAACT | 119 | 58 | qPCR |
|  | R | CCTCCAGGTACTCGTCCTCA |  |  |  |
| MMD | F | GGATCTATGGCATGGGGCTC | 187 | 58 | qPCR |
|  | R | GCTAGAGGTCCAAGCTCACG |  |  |  |
| F4/80 | F | GCACCATCTTGCTGGAGACT | 222 | 60 | qPCR |
|  | R | CTGGGGCCCCTGTAGATACT |  |  |  |
| MHC-Ⅱ | F | CTCGAGGTCATGATCAGCAA | 277 | 60 | qPCR |
|  | R | TGTAAACGTCTCCCCTTTGG |  |  |  |
| β-actin | F | ACCACAGGACTCCATACCCAAGAAAG | 210 | 52-60 | qPCR |
|  | R | GCCGAGAGAGAAATTGTGCGTGAC |  |  |  |

**Supplementary table 2**

**Supplementary table 2 the DEcircRNA-DEmiRNA-DEmRNA networks constructed in this study**

| Up_DEcircRNA | Down_DEmiRNA | Up_DEmRNA |
| --- | --- | --- |
| *novel_circ_0003805* | *gga-miR-458b-5p* | *FAM49A* |
| *novel_circ_0004143* | *gga-miR-132c-5p* | *KCNB2* |
| *novel_circ_0004143* | *gga-miR-132c-5p* | *MMD* |
| *novel_circ_0004143* | *gga-miR-132c-5p* | *DERL1* |
| *novel_circ_0004143* | *gga-miR-132c-5p* | *IFNGR2* |
| *novel_circ_0004143* | *gga-miR-132c-5p* | *EPN2* |
| *novel_circ_0004143* | *gga-miR-132c-5p* | *PHC3* |
| *novel_circ_0004143* | *gga-miR-132c-5p* | *LYN* |
| *novel_circ_0004143* | *gga-miR-132c-5p* | *CCL4* |
| *novel_circ_0004493* | *gga-miR-1729-5p* | *PLA2G15* |
| *novel_circ_0004493* | *gga-miR-1729-5p* | *BCAT1* |
| *novel_circ_0004493* | *gga-miR-1729-5p* | *SPSB1* |
| *novel_circ_0004493* | *gga-miR-1729-5p* | *DENND1B* |
| *novel_circ_0004493* | *gga-miR-1729-5p* | *AFF1* |
| *novel_circ_0004493* | *gga-miR-1729-5p* | *GMFB* |
| *novel_circ_0004433_junction_seq* | *gga-miR-1798-3p* | *NLRC5* |
| *novel_circ_0004433_junction_seq* | *gga-miR-1798-3p* | *FRYL* |
| *novel_circ_0004433_junction_seq* | *gga-miR-1798-3p* | *MMD* |
| *novel_circ_0004433_junction_seq* | *gga-miR-1798-3p* | *ABHD18* |
| *novel_circ_0004433_junction_seq* | *gga-miR-1798-3p* | *C1R* |
| *novel_circ_0004433_junction_seq* | *gga-miR-1798-3p* | *IPPK* |
| *novel_circ_0004433_junction_seq* | *gga-miR-1798-3p* | *MPP7* |
| *novel_circ_0004433_junction_seq* | *gga-miR-1798-3p* | *RAB4A* |
| *novel_circ_0004433_junction_seq* | *gga-miR-1798-3p* | *PTPN2* |
| *novel_circ_0004433_junction_seq* | *gga-miR-1798-3p* | *MFSD11* |
| *novel_circ_0004433_junction_seq* | *gga-miR-1798-3p* | *BCL11B* |
| *novel_circ_0001674* | *gga-miR-1798-3p* | *NLRC5* |
| *novel_circ_0001674* | *gga-miR-1798-3p* | *FRYL* |
| *novel_circ_0001674* | *gga-miR-1798-3p* | *MMD* |
| *novel_circ_0001674* | *gga-miR-1798-3p* | *ABHD18* |
| *novel_circ_0001674* | *gga-miR-1798-3p* | *C1R* |
| *novel_circ_0001674* | *gga-miR-1798-3p* | *IPPK* |
| *novel_circ_0001674* | *gga-miR-1798-3p* | *MPP7* |
| *novel_circ_0001674* | *gga-miR-1798-3p* | *RAB4A* |
| *novel_circ_0001674* | *gga-miR-1798-3p* | *PTPN2* |
| *novel_circ_0001674* | *gga-miR-1798-3p* | *MFSD11* |
| *novel_circ_0001674* | *gga-miR-1798-3p* | *BCL11B* |
| *novel_circ_0001674_junction_seq* | *gga-miR-1798-3p* | *NLRC5* |
| *novel_circ_0001674_junction_seq* | *gga-miR-1798-3p* | *FRYL* |
| *novel_circ_0001674_junction_seq* | *gga-miR-1798-3p* | *MMD* |
| *novel_circ_0001674_junction_seq* | *gga-miR-1798-3p* | *ABHD18* |
| *novel_circ_0001674_junction_seq* | *gga-miR-1798-3p* | *C1R* |
| *novel_circ_0001674_junction_seq* | *gga-miR-1798-3p* | *IPPK* |
| *novel_circ_0001674_junction_seq* | *gga-miR-1798-3p* | *MPP7* |
| *novel_circ_0001674_junction_seq* | *gga-miR-1798-3p* | *RAB4A* |
| *novel_circ_0001674_junction_seq* | *gga-miR-1798-3p* | *PTPN2* |
| *novel_circ_0001674_junction_seq* | *gga-miR-1798-3p* | *MFSD11* |
| *novel_circ_0001674_junction_seq* | *gga-miR-1798-3p* | *BCL11B* |
| *novel_circ_0001674* | *gga-miR-214b-3p* | *ATP2B1* |
| *novel_circ_0001674* | *gga-miR-214b-3p* | *MMD* |
| *novel_circ_0001674* | *gga-miR-214b-3p* | *PHC3* |
| *novel_circ_0001674* | *gga-miR-214b-3p* | *B3GNT5* |
| *novel_circ_0001674* | *gga-miR-214b-3p* | *BCL11B* |
| *novel_circ_0001674* | *gga-miR-214b-3p* | *C25H1ORF43* |
| *novel_circ_0001674* | *gga-miR-214b-3p* | *EHD3* |
| *novel_circ_0001674* | *gga-miR-214b-3p* | *SGK3* |
| *novel_circ_0001674* | *gga-miR-214b-3p* | *IPO7* |
| *novel_circ_0001674* | *gga-miR-214b-3p* | *NBN* |
| *novel_circ_0001674* | *gga-miR-214b-3p* | *IPPK* |
| *novel_circ_0001674* | *gga-miR-214b-3p* | *UBASH3A* |
| *novel_circ_0001674* | *gga-miR-214b-3p* | *DTX3L* |
| *novel_circ_0001674* | *gga-miR-214b-3p* | *PCGF5* |
| *novel_circ_0001674* | *gga-miR-214b-3p* | *MCM2* |
| *novel_circ_0001674* | *gga-miR-214b-3p* | *PTPRE* |
| *novel_circ_0001674* | *gga-miR-214b-3p* | *NUDC* |
| *novel_circ_0001674* | *gga-miR-214b-3p* | *NSMF* |
| *novel_circ_0001674* | *gga-miR-214b-3p* | *ANP32E* |
| *novel_circ_0001674* | *gga-miR-214b-3p* | *44080* |
| *novel_circ_0001674* | *gga-miR-214b-3p* | *BEND3* |
| *novel_circ_0001674* | *gga-miR-214b-3p* | *ABHD17B* |
| *novel_circ_0001674* | *gga-miR-214b-3p* | *STAT1* |
| *novel_circ_0001674* | *gga-miR-214b-3p* | *ABHD18* |
| *novel_circ_0001674* | *gga-miR-214b-3p* | *HIF1A* |
| *novel_circ_0001674* | *gga-miR-214b-3p* | *MOCS1* |
| *novel_circ_0001674* | *gga-miR-214b-3p* | *KCNB2* |
| *novel_circ_0001674* | *gga-miR-214b-3p* | *PSMA1* |
| *novel_circ_0001674* | *gga-miR-214b-3p* | *DNM1L* |
| *novel_circ_0001674* | *gga-miR-214b-3p* | *EHD4* |
| *novel_circ_0001674* | *gga-miR-214b-3p* | *FAM91A1* |
| *novel_circ_0001674* | *gga-miR-214b-3p* | *KCNMA1* |
| *novel_circ_0001674* | *gga-miR-214b-3p* | *TAF4B* |
| *novel_circ_0001674* | *gga-miR-214b-3p* | *EPN2* |
| *novel_circ_0003805* | *gga-miR-30e-3p* | *DENND1B* |
| *novel_circ_0003805* | *gga-miR-30e-3p* | *PHC3* |
| *novel_circ_0003805* | *gga-miR-30e-3p* | *FAS* |
| *novel_circ_0003805* | *gga-miR-30e-3p* | *PDXK* |
| *novel_circ_0003805* | *gga-miR-30e-3p* | *ETV6* |
| *novel_circ_0003805* | *gga-miR-30e-3p* | *C1GALT1* |
| *novel_circ_0003805* | *gga-miR-30e-3p* | *RIF1* |
| *novel_circ_0003805* | *gga-miR-30e-3p* | *RFFL* |
| *novel_circ_0003805* | *gga-miR-30e-3p* | *FAM49A* |
| *novel_circ_0004433* | *gga-miR-30e-3p* | *DENND1B* |
| *novel_circ_0004433* | *gga-miR-30e-3p* | *PHC3* |
| *novel_circ_0004433* | *gga-miR-30e-3p* | *FAS* |
| *novel_circ_0004433* | *gga-miR-30e-3p* | *PDXK* |
| *novel_circ_0004433* | *gga-miR-30e-3p* | *ETV6* |
| *novel_circ_0004433* | *gga-miR-30e-3p* | *C1GALT1* |
| *novel_circ_0004433* | *gga-miR-30e-3p* | *RIF1* |
| *novel_circ_0004433* | *gga-miR-30e-3p* | *RFFL* |
| *novel_circ_0004433* | *gga-miR-30e-3p* | *FAM49A* |
| *novel_circ_0003805* | *gga-miR-458b-5p* | *SNX13* |
| *novel_circ_0003805* | *gga-miR-458b-5p* | *LYN* |
| *novel_circ_0003805* | *gga-miR-458b-5p* | *KCNMA1* |
| down_DEcircRNA | up_DEmiRNA | down_DEmRNA |
| *novel_circ_0003969* | *gga-miR-92-3p* | *RBPMS2* |
| *novel_circ_0003969* | *gga-miR-92-3p* | *SCFD1* |
| *novel_circ_0003969* | *gga-miR-92-3p* | *SDC2* |
| *novel_circ_0003969* | *gga-miR-92-3p* | *TOB1* |
| *novel_circ_0003969* | *gga-miR-92-3p* | *ADAM23* |
| *novel_circ_0003969* | *gga-miR-92-3p* | *ARMC1* |
| *novel_circ_0003969* | *gga-miR-92-3p* | *SH3PXD2A* |
| *novel_circ_0003969* | *gga-miR-92-3p* | *GOLGA3* |
| *novel_circ_0003969* | *gga-miR-92-3p* | *NKX2-3* |
| *novel_circ_0003969* | *gga-miR-92-3p* | *RAB39A* |
| *novel_circ_0003969* | *gga-miR-92-3p* | *TOB2* |
| *novel_circ_0003969* | *gga-miR-92-3p* | *LRRC8C* |
| *novel_circ_0003969* | *gga-miR-92-3p* | *DGKZ* |
| *novel_circ_0003969* | *gga-miR-92-3p* | *TPCN1* |
| *novel_circ_0003969* | *gga-miR-92-3p* | *SSBP3* |
| *novel_circ_0003969* | *gga-miR-92-3p* | *SYT1* |
| *novel_circ_0003969* | *gga-miR-92-3p* | *SRPK2* |
| *novel_circ_0003969* | *gga-miR-92-3p* | *EOGT* |
| *novel_circ_0003969* | *gga-miR-92-3p* | *RRBP1* |
| *novel_circ_0003969* | *gga-miR-92-3p* | *SPHKAP* |
| *novel_circ_0003969* | *gga-miR-92-3p* | *ANO1* |
| *novel_circ_0003969* | *gga-miR-92-3p* | *DPP10* |
| *novel_circ_0003969* | *gga-miR-92-3p* | *KIAA1191* |
| *novel_circ_0003969* | *gga-miR-92-3p* | *LMO2* |
| *novel_circ_0003969* | *gga-miR-92-3p* | *GSAP* |
| *novel_circ_0003969* | *gga-miR-92-3p* | *GUCD1* |
| *novel_circ_0003969* | *gga-miR-92-3p* | *PPCS* |
| *novel_circ_0004752* | *gga-miR-92-3p* | *RBPMS2* |
| *novel_circ_0004752* | *gga-miR-92-3p* | *SCFD1* |
| *novel_circ_0004752* | *gga-miR-92-3p* | *SDC2* |
| *novel_circ_0004752* | *gga-miR-92-3p* | *TOB1* |
| *novel_circ_0004752* | *gga-miR-92-3p* | *ADAM23* |
| *novel_circ_0004752* | *gga-miR-92-3p* | *ARMC1* |
| *novel_circ_0004752* | *gga-miR-92-3p* | *SH3PXD2A* |
| *novel_circ_0004752* | *gga-miR-92-3p* | *GOLGA3* |
| *novel_circ_0004752* | *gga-miR-92-3p* | *NKX2-3* |
| *novel_circ_0004752* | *gga-miR-92-3p* | *RAB39A* |
| *novel_circ_0004752* | *gga-miR-92-3p* | *TOB2* |
| *novel_circ_0004752* | *gga-miR-92-3p* | *LRRC8C* |
| *novel_circ_0004752* | *gga-miR-92-3p* | *DGKZ* |
| *novel_circ_0004752* | *gga-miR-92-3p* | *TPCN1* |
| *novel_circ_0004752* | *gga-miR-92-3p* | *SSBP3* |
| *novel_circ_0004752* | *gga-miR-92-3p* | *SYT1* |
| *novel_circ_0004752* | *gga-miR-92-3p* | *SRPK2* |
| *novel_circ_0004752* | *gga-miR-92-3p* | *EOGT* |
| *novel_circ_0004752* | *gga-miR-92-3p* | *RRBP1* |
| *novel_circ_0004752* | *gga-miR-92-3p* | *SPHKAP* |
| *novel_circ_0004752* | *gga-miR-92-3p* | *ANO1* |
| *novel_circ_0004752* | *gga-miR-92-3p* | *DPP10* |
| *novel_circ_0004752* | *gga-miR-92-3p* | *KIAA1191* |
| *novel_circ_0004752* | *gga-miR-92-3p* | *LMO2* |
| *novel_circ_0004752* | *gga-miR-92-3p* | *GSAP* |
| *novel_circ_0004752* | *gga-miR-92-3p* | *GUCD1* |
| *novel_circ_0004752* | *gga-miR-92-3p* | *PPCS* |
| *novel_circ_0003253* | *gga-miR-223* | *ANO1* |
| *novel_circ_0003253* | *gga-miR-223* | *FAM49A* |
| *novel_circ_0003253* | *gga-miR-223* | *AGAP1* |
| *novel_circ_0003253* | *gga-miR-223* | *MAP1LC3B2* |
| *novel_circ_0003253* | *gga-miR-223* | *KIF3B* |
| *novel_circ_0003253* | *gga-miR-223* | *EZR* |
| *novel_circ_0003253* | *gga-miR-223* | *RCOR3* |
| *novel_circ_0003253* | *gga-miR-223* | *LMO2* |
| *novel_circ_0003253* | *gga-miR-223* | *FGFR2* |
| *novel_circ_0003253* | *gga-miR-223* | *SLC49A3* |
| *novel_circ_0003253* | *gga-miR-223* | *SYBU* |
| *novel_circ_0003253* | *gga-miR-223* | *NTRK2* |
| *novel_circ_0003253* | *gga-miR-223* | *DDX60* |
| *novel_circ_0003253* | *gga-miR-223* | *FAM49A* |
| *novel_circ_0003253* | *gga-miR-223* | *ANKRD17* |
| *novel_circ_0003253* | *gga-miR-223* | *ANO1* |
| *novel_circ_0001305* | *gga-miR-200b-3p* | *PTPN13* |
| *novel_circ_0001305* | *gga-miR-200b-3p* | *PLCL1* |
| *novel_circ_0001305* | *gga-miR-200b-3p* | *TMEM135* |
| *novel_circ_0001305* | *gga-miR-200b-3p* | *TOB1* |
| *novel_circ_0001305* | *gga-miR-200b-3p* | *EDEM3* |
| *novel_circ_0001305* | *gga-miR-200b-3p* | *CYTH3* |
| *novel_circ_0001305* | *gga-miR-200b-3p* | *ZNF652* |
| *novel_circ_0001305* | *gga-miR-200b-3p* | *RIMKLB* |
| *novel_circ_0001305* | *gga-miR-200b-3p* | *GUCD1* |
| *novel_circ_0001305* | *gga-miR-200b-3p* | *TARDBP* |
| *novel_circ_0001305* | *gga-miR-200b-3p* | *SH3PXD2A* |
| *novel_circ_0001305* | *gga-miR-200b-3p* | *TPCN1* |
| *novel_circ_0001305* | *gga-miR-200b-3p* | *SYT1* |
| *novel_circ_0001305* | *gga-miR-200b-3p* | *FAM49A* |
| *novel_circ_0001305* | *gga-miR-200b-3p* | *DACH2* |
| *novel_circ_0001305* | *gga-miR-200b-3p* | *PRDM1* |
| *novel_circ_0001305* | *gga-miR-200b-3p* | *NTRK2* |
| *novel_circ_0001305* | *gga-miR-200b-3p* | *SDC2* |
| *novel_circ_0001305* | *gga-miR-200b-3p* | *GLCCI1* |
| *novel_circ_0001305* | *gga-miR-200b-3p* | *OLFML2A* |
| *novel_circ_0001305* | *gga-miR-200b-3p* | *ELK3* |
| *novel_circ_0001305* | *gga-miR-200b-3p* | *BRSK2* |
| *novel_circ_0001305* | *gga-miR-200b-3p* | *CDR2* |
| *novel_circ_0001305* | *gga-miR-200b-3p* | *DPP10* |
| *novel_circ_0001305* | *gga-miR-200b-3p* | *ABHD18* |
| *novel_circ_0001305* | *gga-miR-200b-3p* | *PCNP* |
| *novel_circ_0001305* | *gga-miR-200b-3p* | *TMEM263* |
| *novel_circ_0001305* | *gga-miR-200b-3p* | *CNTFR* |
| *novel_circ_0001305* | *gga-miR-200b-3p* | *PRDM16* |
| *novel_circ_0001305* | *gga-miR-200b-3p* | *CBX4* |
| *novel_circ_0001305* | *gga-miR-200b-3p* | *PDP1* |
| *novel_circ_0001305* | *gga-miR-200b-3p* | *CNNM2* |
| *novel_circ_0001305* | *gga-miR-200b-3p* | *GSAP* |
| *novel_circ_0001305* | *gga-miR-200b-3p* | *SNX8* |
| *novel_circ_0002663* | *gga-miR-128-1-5p* | *RAP1GAP2* |
| *novel_circ_0002663* | *gga-miR-12247-5p* | *KIF3B* |

**Supplementary table 3**

**Supplementary table 3 the top 20 GO terms enriched by the DEcircRNAs**

| GO_accession | Description | Term_type | Over_represented_pValue |
| --- | --- | --- | --- |
| GO:0043231 | intracellular membrane-bounded organelle | cellular_component | 6.22E-05 |
| GO:0043247 | telomere maintenance in response to DNA damage | biological_process | 6.32E-05 |
| GO:0043227 | membrane-bounded organelle | cellular_component | 6.85E-05 |
| GO:0043229 | intracellular organelle | cellular_component | 0.000292 |
| GO:0043226 | organelle | cellular_component | 0.000325 |
| GO:0006464 | cellular protein modification process | biological_process | 0.000717 |
| GO:0036211 | protein modification process | biological_process | 0.000717 |
| GO:0043412 | macromolecule modification | biological_process | 0.001118 |
| GO:0005634 | nucleus | cellular_component | 0.001142 |
| GO:0044260 | cellular macromolecule metabolic process | biological_process | 0.001193 |
| GO:0006281 | DNA repair | biological_process | 0.001251 |
| GO:0006259 | DNA metabolic process | biological_process | 0.00138 |
| GO:0043170 | macromolecule metabolic process | biological_process | 0.001812 |
| GO:0033326 | cerebrospinal fluid secretion | biological_process | 0.002862 |
| GO:0019538 | protein metabolic process | biological_process | 0.002944 |
| GO:0000266 | mitochondrial fission | biological_process | 0.003024 |
| GO:0006950 | response to stress | biological_process | 0.003189 |
| GO:0061512 | protein localization to cilium | biological_process | 0.003581 |
| GO:0006303 | double-strand break repair via nonhomologous end joining | biological_process | 0.004137 |
| GO:0006289 | nucleotide-excision repair | biological_process | 0.004193 |

**Supplementary table 4**

**Supplementary table 4 the top 20 KEGG pathways enriched by the DEcircRNAs**

| ID | Term | P-Value |
| --- | --- | --- |
| gga03420 | Nucleotide excision repair | 0.005411 |
| gga03450 | Non-homologous end-joining | 0.036377 |
| gga05164 | Influenza A | 0.053726 |
| gga05168 | Herpes simplex infection | 0.063438 |
| gga03022 | Basal transcription factors | 0.102782 |
| gga00510 | N-Glycan biosynthesis | 0.125592 |
| gga04622 | RIG-I-like receptor signaling pathway | 0.147842 |
| gga04350 | TGF-beta signaling pathway | 0.181374 |
| gga04070 | Phosphatidylinositol signaling system | 0.195353 |
| gga04912 | GnRH signaling pathway | 0.199961 |
| gga04540 | Gap junction | 0.206826 |
| gga04620 | Toll-like receptor signaling pathway | 0.213634 |
| gga04114 | Oocyte meiosis | 0.224855 |
| gga04270 | Vascular smooth muscle contraction | 0.251162 |
| gga03040 | Spliceosome | 0.261867 |
| gga04910 | Insulin signaling pathway | 0.289016 |
| gga04530 | Tight junction | 0.289016 |
| gga04120 | Ubiquitin mediated proteolysis | 0.30122 |
| gga04630 | Jak-STAT signaling pathway | 0.303234 |
| gga04020 | Calcium signaling pathway | 0.373952 |

**Supplementary table 5**

**Supplementary table 5 the top 20 GO terms enriched by the DEmiRNAs**

| GO_accession | Description | Term_type | Over_represented_pValue |
| --- | --- | --- | --- |
| GO:0001071 | nucleic acid binding transcription factor activity | molecular_function | 3.47E-09 |
| GO:0003700 | transcription factor activity, sequence-specific DNA binding | molecular_function | 3.47E-09 |
| GO:0000975 | regulatory region DNA binding | molecular_function | 3.46E-08 |
| GO:0001067 | regulatory region nucleic acid binding | molecular_function | 3.46E-08 |
| GO:0044212 | transcription regulatory region DNA binding | molecular_function | 3.46E-08 |
| GO:0006366 | transcription from RNA polymerase II promoter | biological_process | 3.93E-08 |
| GO:0006357 | regulation of transcription from RNA polymerase II promoter | biological_process | 4.05E-08 |
| GO:0007399 | nervous system development | biological_process | 2.84E-07 |
| GO:0000976 | transcription regulatory region sequence-specific DNA binding | molecular_function | 4.23E-07 |
| GO:0030182 | neuron differentiation | biological_process | 4.88E-07 |
| GO:0043565 | sequence-specific DNA binding | molecular_function | 8.36E-07 |
| GO:0000981 | RNA polymerase II transcription factor activity, sequence-specific DNA binding | molecular_function | 9.37E-07 |
| GO:0001012 | RNA polymerase II regulatory region DNA binding | molecular_function | 4.80E-06 |
| GO:0010468 | regulation of gene expression | biological_process | 5.76E-06 |
| GO:0006355 | regulation of transcription, DNA-templated | biological_process | 6.27E-06 |
| GO:1903506 | regulation of nucleic acid-templated transcription | biological_process | 8.88E-06 |
| GO:2001141 | regulation of RNA biosynthetic process | biological_process | 8.88E-06 |
| GO:0000977 | RNA polymerase II regulatory region sequence-specific DNA binding | molecular_function | 9.20E-06 |
| GO:0022008 | neurogenesis | biological_process | 1.06E-05 |
| GO:0006351 | transcription, DNA-templated | biological_process | 1.20E-05 |

**Supplementary table 6**

**Supplementary table 6 the top 20 KEGG pathways enriched by the DEmiRNAs**

| Pathway_term | P-Value |
| --- | --- |
| Intestinal immune network for IgA production | 0.003503 |
| Notch signaling pathway | 0.004602 |
| Salmonella infection | 0.035502 |
| Calcium signaling pathway | 0.038652 |
| Phenylalanine metabolism | 0.04175 |
| Cell adhesion molecules (CAMs) | 0.059811 |
| Lysine degradation | 0.061167 |
| Other glycan degradation | 0.078013 |
| Influenza A | 0.079057 |
| Galactose metabolism | 0.101482 |
| Selenocompound metabolism | 0.110337 |
| TGF-beta signaling pathway | 0.113496 |
| Ribosome | 0.141481 |
| beta-Alanine metabolism | 0.141632 |
| Steroid hormone biosynthesis | 0.144572 |
| Herpes simplex infection | 0.168547 |
| Cardiac muscle contraction | 0.171007 |
| Oxidative phosphorylation | 0.186108 |
| Riboflavin metabolism | 0.189984 |
| FoxO signaling pathway | 0.196324 |

**Supplementary table 7**

**Supplementary table 7 the top 20 GO terms enriched by the DEmRNAs**

| GO_accession | Description | Term_type | Over_represented_pValue |
| --- | --- | --- | --- |
| GO:0006955 | immune response | biological_process | 1.03E-12 |
| GO:0002376 | immune system process | biological_process | 1.05E-11 |
| GO:0002252 | immune effector process | biological_process | 3.40E-09 |
| GO:0002682 | regulation of immune system process | biological_process | 4.36E-09 |
| GO:0050776 | regulation of immune response | biological_process | 4.58E-09 |
| GO:0019221 | cytokine-mediated signaling pathway | biological_process | 3.35E-08 |
| GO:0048584 | positive regulation of response to stimulus | biological_process | 6.00E-08 |
| GO:0002684 | positive regulation of immune system process | biological_process | 9.45E-07 |
| GO:0009967 | positive regulation of signal transduction | biological_process | 1.03E-06 |
| GO:0010647 | positive regulation of cell communication | biological_process | 1.14E-06 |
| GO:0023056 | positive regulation of signaling | biological_process | 1.58E-06 |
| GO:0071345 | cellular response to cytokine stimulus | biological_process | 1.61E-06 |
| GO:0070013 | intracellular organelle lumen | cellular_component | 1.88E-06 |
| GO:0031974 | membrane-enclosed lumen | cellular_component | 1.97E-06 |
| GO:0043233 | organelle lumen | cellular_component | 2.02E-06 |
| GO:0051707 | response to other organism | biological_process | 2.87E-06 |
| GO:0044428 | nuclear part | cellular_component | 3.37E-06 |
| GO:0034097 | response to cytokine stimulus | biological_process | 3.50E-06 |
| GO:0043231 | intracellular membrane-bounded organelle | cellular_component | 3.70E-06 |
| GO:0048518 | positive regulation of biological process | biological_process | 4.02E-06 |

**Supplementary table 8**

**Supplementary table 8 the top 20 KEGG pathways enriched by the DEmRNAs**

| ID | Term | P-Value |
| --- | --- | --- |
| gga04630 | Jak-STAT signaling pathway | 0.000641 |
| gga04060 | Cytokine-cytokine receptor interaction | 0.00223 |
| gga05168 | Herpes simplex infection | 0.005513 |
| gga03010 | Ribosome | 0.005891 |
| gga04620 | Toll-like receptor signaling pathway | 0.02735 |
| gga04144 | Endocytosis | 0.033923 |
| gga00790 | Folate biosynthesis | 0.043411 |
| gga03060 | Protein export | 0.048304 |
| gga04142 | Lysosome | 0.052436 |
| gga00770 | Pantothenate and CoA biosynthesis | 0.059954 |
| gga00860 | Porphyrin and chlorophyll metabolism | 0.067731 |
| gga03018 | RNA degradation | 0.081236 |
| gga04672 | Intestinal immune network for IgA production | 0.125962 |
| gga00250 | Alanine, aspartate and glutamate metabolism | 0.125962 |
| gga04122 | Sulfur relay system | 0.133486 |
| gga05164 | Influenza A | 0.137539 |
| gga04115 | p53 signaling pathway | 0.150279 |
| gga04150 | mTOR signaling pathway | 0.190862 |
| gga01210 | 2-Oxocarboxylic acid metabolism | 0.191506 |
| gga00020 | Citrate cycle (TCA cycle) | 0.231483 |
